# Sperm kinetics of Egyptian buffalo bulls (*Bubalus bubalis*) affected by the red laser postfreezing

**DOI:** 10.5455/javar.2022.i607

**Published:** 2022-09-30

**Authors:** Sherif Mohamed Dessouki, Dalia Abd-El Rahman Ahmed, Ayat Kassem Fayed

**Affiliations:** Department of Animal Production, Faculty of Agriculture, Cairo University, Cairo, Egypt

**Keywords:** Buffalo, freezing, motility, sperm red laser

## Abstract

**Objective:**

To improve the fertilizing ability of frozen buffalo semen using the more beneficial, accurate, and cheap technique of laser irradiation at specific wavelengths and exposure times.

**Materials and Methods:**

The red laser source (625 nm) was used in this study with 5 watts output power and for the irradiation of the semen samples for 5 min; the laser focus spot area was 1 cm^2^.Thirty straws belonging to five buffalo bulls were used in this study.

**Results:**

The results show that total motility (%) and progressive motility (%) increased insignificantly after 5 min of exposure (73.8 ± 1.4 and 60.4 ± 1.1, respectively) compared to the control sample (70.9 ± 0.9 and 57.5 ± 1.7, respectively). All velocity parameters (velocity average path,velocity curved line, and velocity straight line µm/sec) recorded a significant (p < 0.05) increase in samples measured 5 min after exposure (52.3 ± 1.3, 83.5 ± 2.0, and 43.5 ± 1.2, respectively) compared to the untreated ones (47.1 ± 2.0, 76.3 ± 3.1, and 38.6 ± 1.9, respectively).

**Conclusion:**

The application of the red laser light on buffalo semen postthawing resulted in a positive correlation with almost every motility parameter; it may be recommended to apply this technique pre-in vitro fertilization for embryo production of buffalo species.

## Introduction

The artificial insemination (AI) tool is widely used today for all mammalian species. Freezing semen hurts the survival and fertilization capacity, leading to sperm damage (cryoinjuries), destabilization of the plasmatic membrane, consequent elevation of the intracellular calcium concentration, poor fertility potential, increasing oxidative stress, and decreasing pregnancy rates [1–3]. It is very essential to fulfill the monitoring of cryopreserved semen quality to conjure up an image of its implied fertility.

Many techniques have been used to increase the freezing and fertilizing abilities of semen after thawing. One of the most sensitive and least costly techniques used with mammalian species is semen exposure to different types of laser irradiation. Previous studies have reported that exposure to laser irradiation increases the percentage of live sperm cells and improves sperm motility by increasing energy supply and consumption, besides intensifying fertilizing capacity for Friesian bulls [4,5], donkeys [6], and stallions [7].

Fernandes et al.[8] demonstrated that low-laser radiation has been used to increase the tissue repair rate, and it was applied for inflamed tissue and damaged cell healing. Furthermore, semen characteristics improvement by using laser irradiation in vitro experiments was reported by Firestone et al. [9] and Preece et al. [10], who found semen samples that were exposed to low-level laser light were enhanced in their motion traits and protected the DNA of the cryopreserved cell from damage. The laser light benefitting the sperm cells during cryopreservation conditions could be summarized by the elimination of superoxide anions that can increase the hyperactivation of the sperm cells and reduce superoxide dismutase enzyme activity. Finally, exposure of sperm cells to laser light protects them from the deleterious effect of ROS production. The effect of the laser light on the DNA of bovine sperm during cryopreservation has been studied by Fernandes et al. [8]. This impact was reported in many species: humans, rabbits, buffaloes, dogs, and turkeys. Corral-Baqués et al. [11] reported that exposure of dogs’ sperm to laser light at 655 nm improved the characteristics of semen in addition to the plasma membrane integrity and the intactness of the acrosomal membrane. Application of low-level helium–neon (He-Ne) laser on bovine semen has been revealed by Siqueira et al.[12], who found an enhancement in sperm motion characteristics and sperm velocity. Moreover, the increase in sperm viability during cryopreservation resulted from the activation of the respiratory chain after the absorption and the interaction of light photons and interaction with photoreceptors or chromophores. Furthermore, the usefulness of irradiation on cryopreserved sperm cells based on the wavelength, type, power, and dose of laser light was demonstrated previously [13,4], showing more benefits by using low levels with shorter wavelengths of a laser, which were absorbed more by the sperm cells during cryopreservation.

Another role of the low-intensity laser light was reported by Miktadova et al.[14], who reported on the role of low-intensity laser light on human semen, suggesting that laser irradiation activates functional genes that prevent cell nucleic acid from being damaged. Furthermore, using laser light and holographic analysis helps measure sperm kinematic parameters and offers the possibility of avoiding intrinsic sperm movement, which causes an artificial motility pattern [15].

Recent research has demonstrated that red light stimulation improves sperm motility, the ability to trigger in vitro capacitation, and the fertilizing ability of fresh and frozen–thawed sperm [7]. The application of laser light beams at low energy increases mitochondrial activity and ATP synthesis. It changes the cell’s redox state in human, mouse, dog, bull, sheep, and rabbit sperm [16].

Computer-assisted semen analysis (CASA) examined many sperms quickly, giving an assemblage of quantitative data on kinematics and sperm morphology, so it can probably optimize the accuracy of seminal analyses [17]. Patel and Dhami [18] and Carvalho et al.[19] reported a direct correlation between the motility characteristics of buffaloes (Bubalus bubalis) and the fertilizing ability of their sperm cells. The CASA software is applied to measure the fine changes in motion characteristics of sperm and, subsequently, their potential to achieve the egg cell’s fertilization process successfully [20]. This work aimed to find out what happens when a red laser shines on Egyptian buffalo sperm after it has been thawed.

## Materials and Methods

### Ethical approval

The semen samples were obtained from a semen bank that belongs to the AI Lab Faculty of Agriculture at Cairo University. So, there was no direct contact (or use) with sires. In addition, the sires passed away in 2009, and before the establishment of the IUACVC committee, they were working. So, ethical approval was not required at this time.

### Buffalo bulls and semen samples

The study used frozen semen samples from five healthy and sexually mature, and active Egyptian buffaloes (B. bubalis). The bulls were 3–5 years old at the time of semen collection, with an average body weight of 592 ± 55 kg. The animal selection criteria included buffalo bulls, which were healthy and free of diseases and parasites. Semen samples were diluted in a Tris-based extender (3.61 gm tris-hydroxymethyl aminomethane, 1.89 gm citric acid, 20 ml egg yolk, 20 ml glycerol, 100,000 IU Penicillin G sodium, 50,000 IU streptomycin sulfate, and bi-distilled water up to 100 ml). The buffalo semen straws were cryopreserved in liquid nitrogen until the beginning of the study.

### Laser source and irradiation procedure

The red laser source (625 nm) was used in this study with 5 watts of output power and for the irradiation of the semen samples for 5 min; the laser focus spot area was 1 cm^2^, and for the stability of the laser stream, the device was switched on before the start of the experiment for testing.

### Software analysis (CASA)

The software used is the CASA sperm analyzer (Sperm Visionminitube Hauptstrae 41. 84184 Tiefenbach, Germany), which is used to provide live images by the computer-assisted microscope (Olympus), and evaluation of sperm motion characteristics. The motion characterization was recorded, including distance curved line (DCL, µm), distance average path (DAP, µm), distance straight line (DSL, µm), velocity curved line (VCL, µm/sec), velocity average path (VAP, µm/sec), velocity straight line (VSL, µm/sec), linearity (LIN = VSL/VCL), straightness (STR = VSL/VAP), wobble (WOB = VAP/VCL), beat cross frequency (BCF, Hz), and amplitude of lateral head displacement (ALH, µm).

### Sperm sample preparation

**Figure 1.**
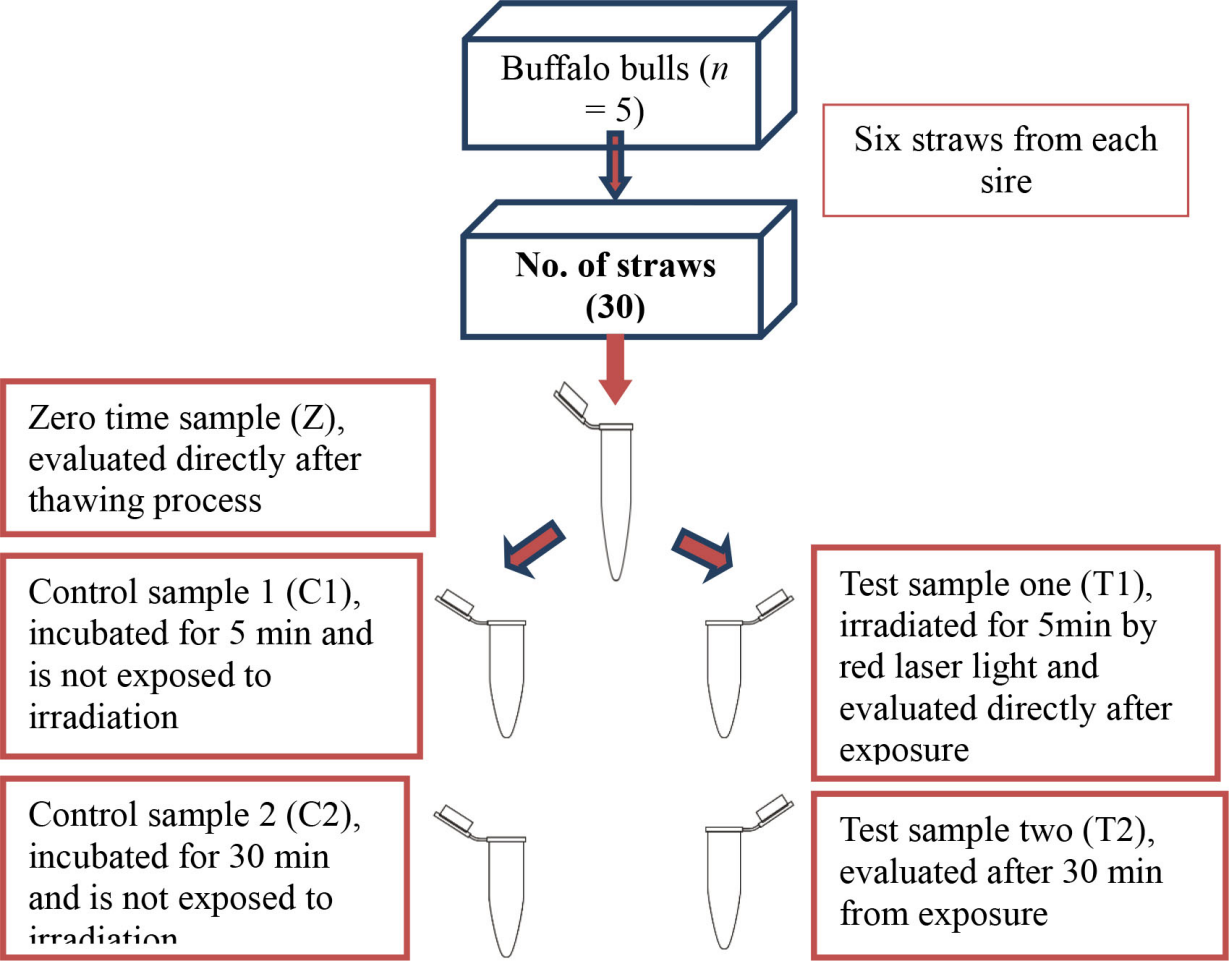
Scheme for the exposure of semen samples to the red laser.

The French plastic straws were stored at −196°C. The straws were removed from the liquid nitrogen tank and placed in a water bath preheated to 39°C for 30 sec. The content of each straw was then emptied into a 1.5 ml Eppendorf tube, labeled, and placed back into the water bath at 37°C for complete thawing of the sample. All steps were carried out in sterile and shaded conditions to maintain the current state of the semen samples and not to allow any contact with any other light source before exposure. A total of 30 straws were used in this study based on using a pool of postthawed semen from 2 French straws for each sire, with 3 replicates for each bull. After the thawing process of the semen samples, 200 µl aliquots were taken from the main Eppendorf and put into 5 Eppendorf. The Eppendorf from a single sire was split into five groups (Fig. 1):

- Z (Zero time sample; the initial evaluation of the semen sample directly after the thawing process);
- T1 (Test sample one; irradiated for 5 min by the red laser light and evaluated directly after exposure)
- C1 (Control sample one; evaluated after 5 min of incubation);
- T2 (Test sample two; evaluated after 30 min of exposure);
- C2 (Control sample two; evaluated after 30 min of incubation).

Three replicates from each sire were used for the study. For each observation, two straws were extracted from the liquid nitrogen tank, thawed, and divided into the five groups for exposure and evaluation. The samples were handled under sterile conditions and were kept in dark conditions for the duration of the incubation until the time of evaluation. The evaluation was accomplished with 2,000 sperm cells for each sample.

### Statistical analysis

The motion characteristics data produced from the evaluation of the semen samples and irradiated by the red laser light from the five sires were analyzed using the SAS GLM procedure [21]. The analysis was carried out to determine the impacts of the red laser light (625 wavelengths) on the measured traits by using the following formula: *Y* = *u* + *E*_i_ + *T*_j_ + (*E*_i_ × *T*_j_)_k_+ *e*_ijkl_, *Y* = measured trait, *u* = overall mean, *E*i = effect of the red laser light, *T*_j_ = effect of time, (*E*_i_ × *T*_j_)_k_ = interaction between the effect of laser and effect of time, and *e*_ijkl_ = experimental error. All the data used in the statistical analysis were retrieved from the CASA databank.

## Results

**Figure 2.**
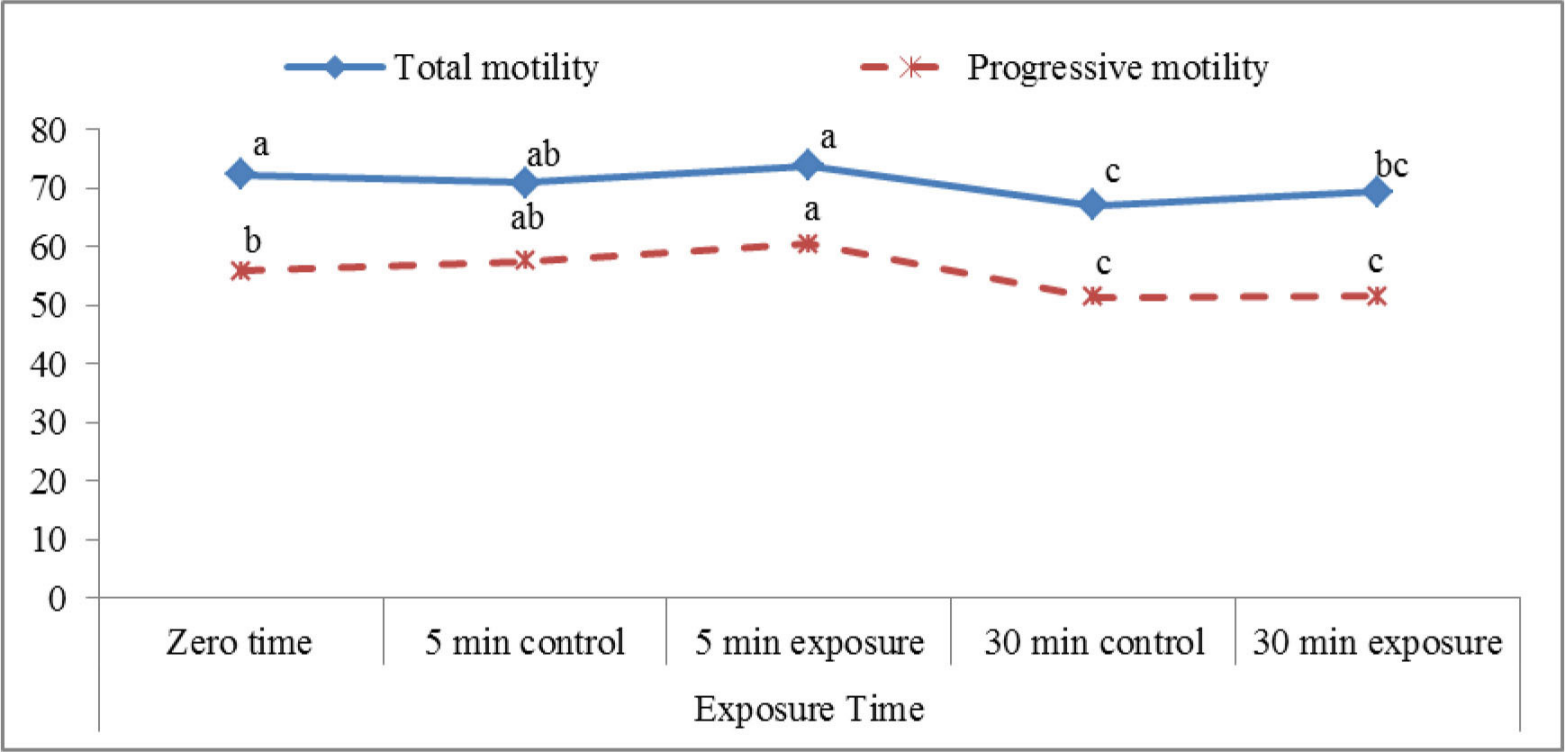
Total and progressive motility percentages affected by exposure to the red laser.

This experiment was designed to study the effect of red laser irradiation as a simple approach to improve sperm motion characteristics and increase the fertilizing ability of postthawed semen for in vitro applications. The results of total and progressive motility (Fig. 2) showed significant differences (p < 0.05) between control and exposed samples. Total and progressive motility percentages 5 min after exposure to the red laser revealed a significant increase (73.8 ± 1.4 and 60.4 ± 1.1, respectively) in comparison to control samples (70.9 ± 0.9 and 57.5 ± 1.7, respectively). On the other hand, incubation of semen samples for 30 min leads to a significantly higher percentage of total motility (69.4 ± 0.9) compared to the control (67.1 ± 1.1). In this study, the length of time in the incubator did not help the treated samples move forward.

**Figure 3.**
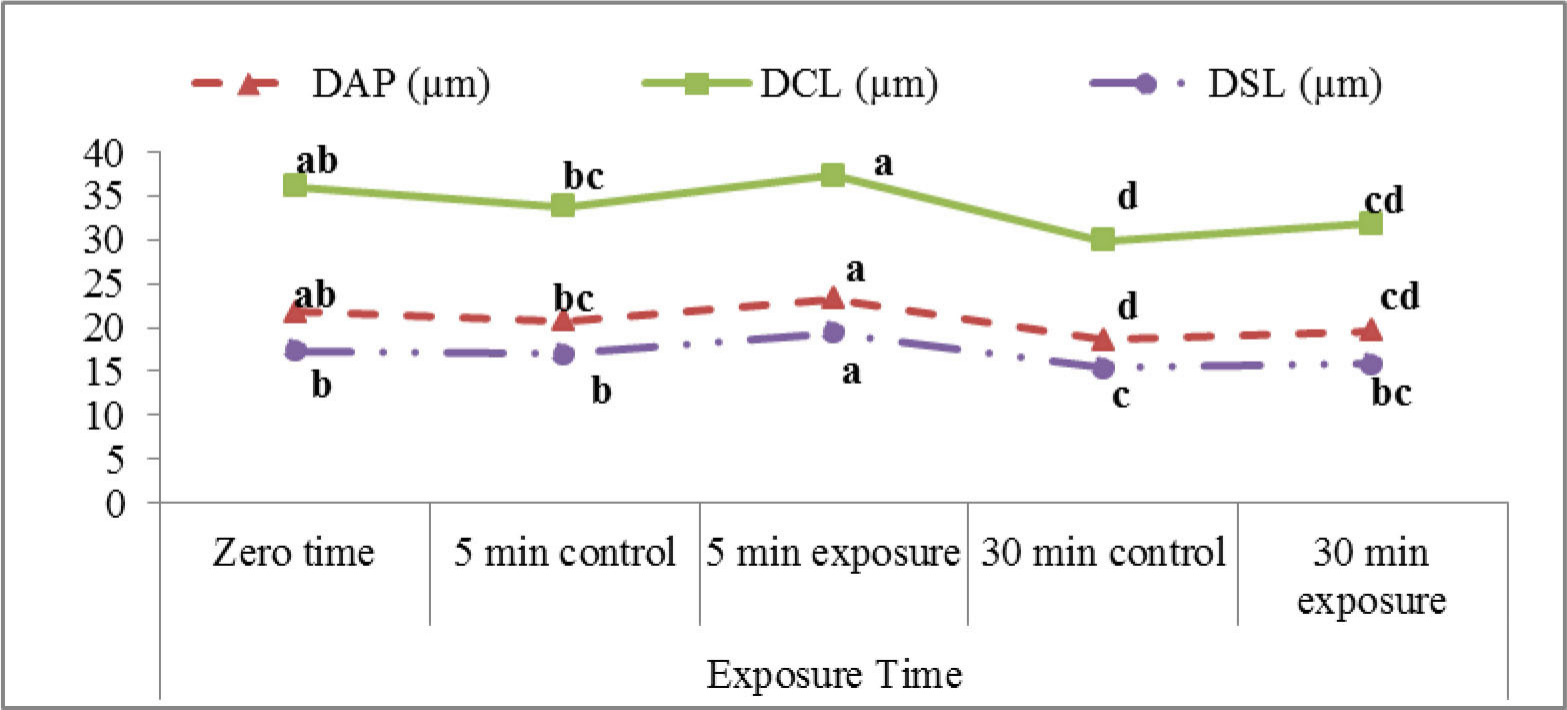
Distance average path (DAP; µm), distance curved line (DCL; µm), and distance straight line (DSL, µm) of buffalo semen affected by exposure to the red laser.

The obtained results in this study of DAP and DSL (µm) indicated a significant increase (p < 0.05) 5 min after exposure (23.3 ± 0.5 and 19.4 ± 0.5, respectively) in comparison to control samples (20.8 ± 0.8 and 17.0 ± 0.8, respectively). On the other hand, incubation revealed an insignificant difference between exposed and unexposed samples (Fig. 3). DCL was significantly higher 5 and 30 min after the red laser exposure (37.4 0.8 and 31.9 1.0 m, respectively) than control (33.8 1.3 and 29.9 0.9 µm, respectively).

**Figure 4.**
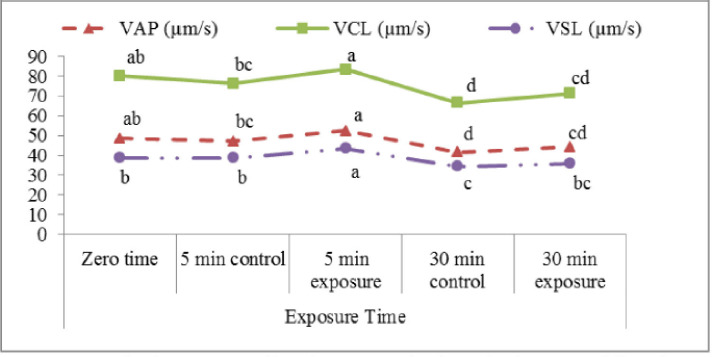
Velocity average path (VAP; µm/sec), velocity curved line (VCL; µm/sec), and velocity straight line (VSL; µm/sec) affected by exposure to the red laser.

The effect of the red laser on sperm VAP, VCL, and VSL of buffalo semen (Fig. 4) was not significant after 30 min of incubation. However, there was a slight increase in these rates (44.2 ± 1.5, 71.4 ± 2.4, and 35.9 ± 1.5 µm/sec, respectively) compared to control samples (41.6 ± 1.3, 66.5 ± 2.1, and 34.4 ± 1.3 µm/sec, respectively). On the other hand, sperm velocity parameters (VAP, VCL, and VSL) were significantly higher (p < 0.05) after 5 min of exposure to the red laser (52.3 ± 1.3, 83.5 ± 2.0 and 43.5 ± 1.2 µm/sec, respectively) than unexposed samples (47.1 ± 2.0, 76.3 ± 3.1, and 38.6 ± 1.9 µm/sec, respectively).

**Figure 5.**
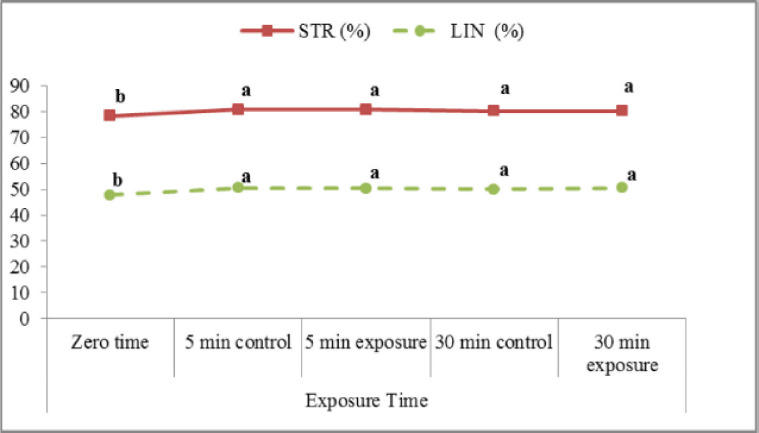
Straightness (STR; VSL/VAP) and linearity (LIN; VSL/VCL) affected by exposure to the red laser.

**Figure 6.**
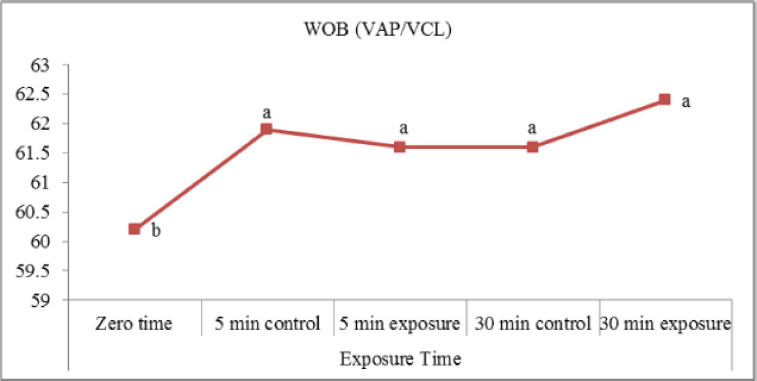
Wobble (WOB; VAP/VCL) affected by exposure to the red laser.

**Figure 7.**
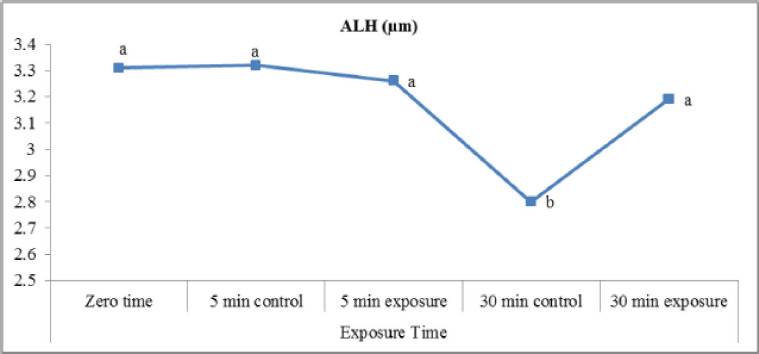
ALH affected by exposure to the red laser.

**Figure 8.**
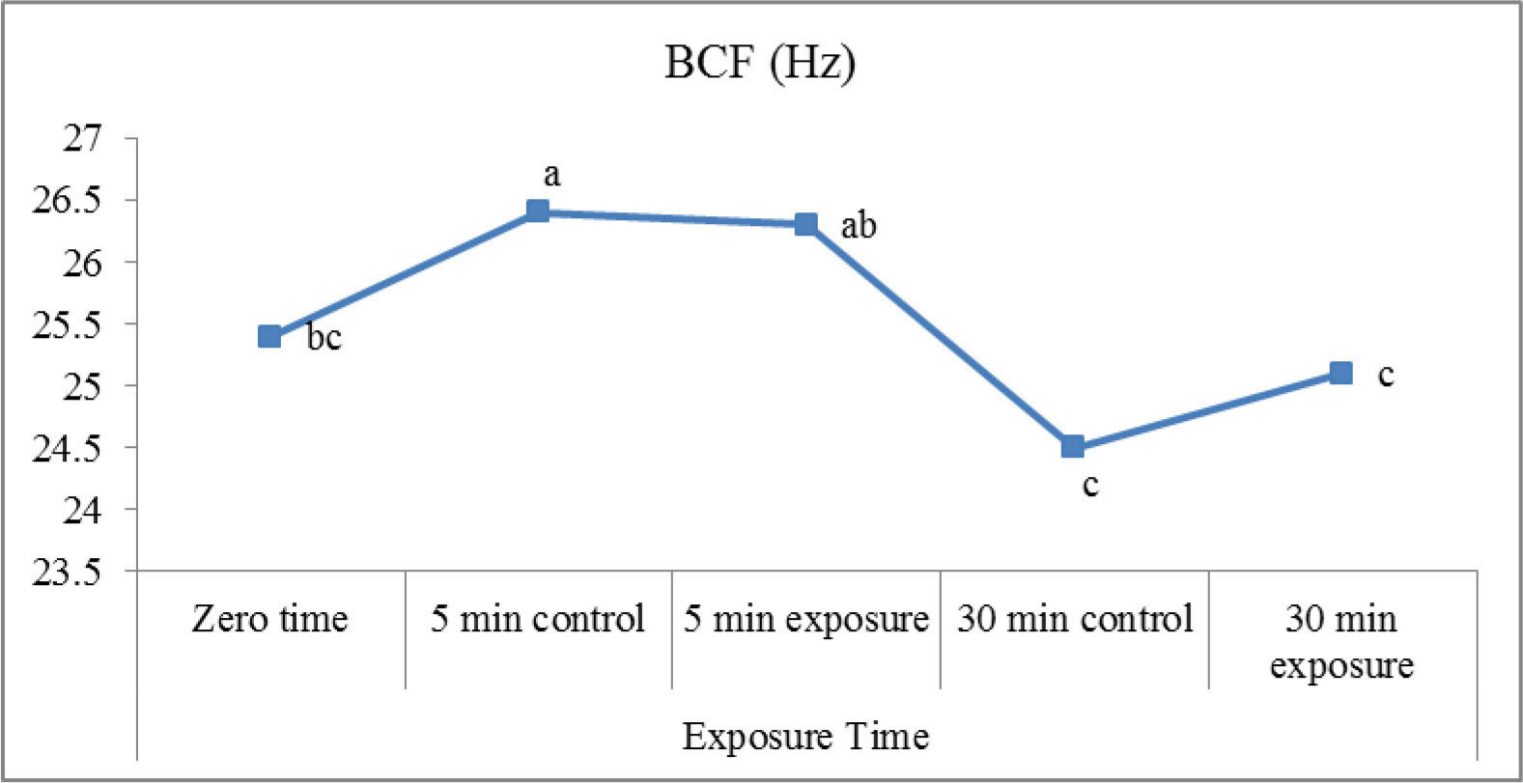
Beat cross frequency (BCF) (Hz) affected by exposure to the red laser.

The obtained results (Fig. 5) indicated an insignificant difference between exposed and control samples (indicating that there were no significant differences) regarding LIN% (VSL/VCL) and STR% (VSL/VAP). A slight increase in LIN (%) in exposed samples postincubation for 30 min (50.4 ± 0.6) is compared to the control (49.9 ± 0.5). In the present study (Fig 6), exposure of samples to red laser irradiation did not affect the WOB (VAP/VCL) percentage. Moreover, the incubation period had no significant effect on this trait in treated and control samples. The results of ALH (Fig 7) revealed a slight, insignificant decrease in exposed samples (3.26 ± 0.07 µm) compared to control (3.32 ± 0.11 µm). On the other hand, incubated samples in control were significantly lower (2.80 ± 0.07 µm) than treated ones (3.19 ± 0.08 µm). Red laser irradiation did not affect BCF (Hz) (Fig. 8) of frozen buffalo sperm either preincubation or postincubation.

## Discussion

This experiment was designed to investigate the effect of exposure to red laser (625 nm) with 5 watts of irradiation on frozen buffalo spermatozoa motion characteristics. Some sperm parameters were improved after exposure to irradiation compared to control (i.e., %total motility, %progressive motility, VCL, VAP, and VSL). Our current study defends the recommended chemical mechanism of intracellular photonic absorption, implying that the photonic energy in the red light is absorbed by cytochrome-c oxidase, increasing ATP production and thus leading to improved sperm motility [10]. Previous reports indicate that laser irradiation of bull sperm may stimulate Ca2++ exchange through the cell membrane and increase nitric oxide formation [22]. In agreement with the findings in this study, Catalán et al. [7] reported on stallion semen by using irradiation of the red laser at a wavelength of 620–630 nm with an exposure pattern (light: 3 min, dark: 3 min, light: 3 min) and an increase in some motion characteristics, mitochondrial membrane potential, and intracellular ROS without affecting the sperm viability or the integrities of the plasma membrane and acrosome. Furthermore, Yeste et al. [23] discovered that exposing boar sperm to red lasers with different wavelengths ranging from 620 to 630 nm improved most motility parameters while having no effect on sperm viability or acrosome integrity.

The same results for bovine semen kinetic analysis were obtained by Carvalho et al. [19] using red and green laser illuminations at 632 and 532 nm, respectively, who found a positive correlation between both types of laser and sperm velocity in addition to percent sperm cell motility. Moreover, Iaffaldano et al. [24] mentioned that He-Ne laser irradiation at 6.12 J/cm2 enhanced ram cryopreserved semen quality, mass sperm motility, ATP content, progressive motility, and viability.

The results of the current research concurred with the results of Gabel et al. [25]. They demonstrated that by using two sources of low laser irradiation gallium–aluminum–arsenide (GaAlAs) single laser (810 nm; 200 mW) and LED cluster (660 and 850 nm) on fresh and frozen human semen samples, the sperm motility index and total functional sperm count increased fourfold compared to nonexposure samples. Moreover, Siqueira et al. [12] found that the exposure of a He-Ne laser at a low level (633 nm) to buffalo sperm postthawing for 5 min increased the VAP of the treated sample by an average of 98.48 µm/sec, compared to 83.48 µm/sec for the untreated samples. Moreover, the curvilinear velocity was reported at 189.2 µm/sec for the exposed samples compared to unexposed samples at 159.28 µm/sec. Almeida et al. [16] used a GaAlAs laser device with an 808-nm infrared wavelength on cryopreserved sperm from White Dorper rams. They found that low-level laser therapy had no clear effect on improving sperm quality from rams that had their testicles insulated.

In frozen–thawed human semen samples, Preece et al. [10] used a wavelength of 633 nm and recorded the curvilinear velocity (VCL) to be higher than the control group. All samples exposed to the laser exhibited higher average curvilinear velocity (VCL) measurements than their control counterparts by 17%–47%. Firestone et al. [9] recorded that the motility of the treated sample for 30 sec by laser light at a low level improved by 17.1% at 905 nm wavelength compared to untreated semen samples. Fernandes et al. [8] applied a short (660 nm) wavelength and 30 mW power of an aluminum–gallium–indium–phosphide laser diode on postthawed bovine semen and reported an increase in progressive motility of 43.5 % ± 7.7% in irradiated samples in comparison to control (30.4% ± 10.5%). Abdel-Salam et al. [13] detected an enhancement in sperm traits such as motility, progressive sperm velocity parameters (VAP, VCL, and VSL), and distance parameters (DAP, DCL, and DSL) when utilizing the 532 nm wavelength of the green laser on buffalo semen.

By using different time intervals in dogs’ semen by Corral-Baqués et al. [11], who found that the velocity parameters (VAP, VCL, and VSL) were increased when exposed to a continuous wave diode laser for 45 min. On the other hand, postthawed human semen showed improvement with a short time of exposure to the red laser, as mentioned by Preece et al. [10] for the average motion traits. Siqueira et al. [12] recorded the effect of the He-Ne laser treatment on motion characteristics, ATP production, velocity traits, and metabolic function of semen and found an increase in motion traits (total and progressive motility) after exposure and incubation for 25 min. As reported in the buffalo semen [13], motion traits, velocity characteristics (VAP, VCL, and VSL) enhancement, and lower (ALH) were reported after exposure to the green laser at 532 nm wavelength irradiation for 10 min, which is in agreement with this study. Furthermore, the exposure of sperm cells to laser irradiation resulted in an increase in the hyperactivation of sperm cells, which could be measured in the elevated rates of VCL, VSL, and LIN percentages and the decreased value of ALH. Yazdi et al. [26] applied laser light that ranged from 400 to 800 nm wavelength and recorded a 40% increase in hyperactivated cells compared to untreated human semen samples.

In the present study, postfrozen and thawed samples of Egyptian buffalo bulls were exposed to the red laser light (625 nm) wavelength and examined for motion characteristics by using CASA. Motility traits were recorded 5 and 30 min after exposure to evaluate the effect of the incubation period after irradiation. The irritated semen samples revealed a significant increase in motility traits (progressive motility) immediately after exposure. Furthermore, the velocity traits (VSL, VAP, and VCL), BCF, STR, and LIN percentages in irradiated semen samples recorded higher values than the control samples. The present results agree with Corral-Baqués et al. [11] and Preece et al. [10]. On the other hand, the ALH value of the control samples was higher than that of the samples that had been exposed to the red light.

Application of red laser irradiation on buffalo sperm has an obvious effect on the average value of BCF when compared to control semen samples. The results contradict those of Abdel-Salam et al. [13] on the same species when using the green light of laser irradiation. Meanwhile, the findings of Fernandes et al. [8] on bovine semen postfreezing using a low-level laser (660 nm) wavelength agree with the present study.

The results of the present work indicated higher values of VCL (83.5 μm/sec), ALH (3.2 μm), LIN (50.3%), and BCF (26.3 Hz) than the findings of Fernandes et al. [8] on bovine semen (80 μm/sec, 2.8 μm, 50.3%, and 10 Hz, respectively), which reflect the beneficial effect of the red laser light on sperm motion characteristics postcryopreservation in Egyptian buffalo bulls.

## Conclusion

In conclusion, sperm kinetics (motion characteristics and velocity parameters) of Egyptian buffalo bulls display positive results when exposed to a 625 nm wavelength of the red laser light, either after 5 min or after 30 min of exposure. The difference in the period of incubation postirradiation has a clear impact on the results. As demonstrated, red laser light irradiation is a great tool for improving sperm motility and velocity characteristics to increase fertilization rates during *in vitro* fertilization (IVF). Further studies are required to determine the efficiency of sperm in IVF using the exposed samples and the changes that may occur at the molecular level from laser treatment.

## List of Abbreviations

AI, Artificial insemination; ALH, Amplitude of lateral head displacement; BCF, Beat cross frequency; CASA, Computer-assisted semen analysis; DAP, Distance average path; DCL, Distance curved line; DSL, Distance straight line; IVF, *In vitro* fertilization; LIN, Linearity percentage; ROS, Reactive oxygen species; ATP, Adenosine triphosphate; GLM, General linear model; SAS, Statistical Analysis System; LED, Light-emitting diode; VAP, Velocity average line; VCL, Velocity curved path; VSL, Velocity straight line µm/sec; WOB, Wobble motion.
